# A generalized Robinson-Foulds distance for labeled trees

**DOI:** 10.1186/s12864-020-07011-0

**Published:** 2020-11-18

**Authors:** Samuel Briand, Christophe Dessimoz, Nadia El-Mabrouk, Manuel Lafond, Gabriela Lobinska

**Affiliations:** 1 Computer Science Department, Université de Montréal, Montreal, Canada; 2 Department of Computational Biology, University of Lausanne, Lausanne, Switzerland; 3 Department of Genetics Evolution and Environment, University College London, London, UK; 4 Center for Integrative Genomics, University of Lausanne, Lausanne, Switzerland; 5 Swiss Institute of Bioinformatics, Lausanne, Switzerland; 6 Department of Computer Science, University College London, London, UK; 7 Computer Science Department, Université de Sherbrooke, Sherbrooke, Canada

**Keywords:** Edit distance, Labeled trees, Robinson-Foulds, Tree metric

## Abstract

**Background:**

The Robinson-Foulds (RF) distance is a well-established measure between phylogenetic trees. Despite a lack of biological justification, it has the advantages of being a proper metric and being computable in linear time. For phylogenetic applications involving genes, however, a crucial aspect of the trees ignored by the RF metric is the type of the branching event (e.g. speciation, duplication, transfer, etc).

**Results:**

We extend RF to trees with labeled internal nodes by including a node *flip* operation, alongside edge contractions and extensions. We explore properties of this extended RF distance in the case of a binary labeling. In particular, we show that contrary to the unlabeled case, an optimal edit path may require contracting “good” edges, i.e. edges shared between the two trees.

**Conclusions:**

We provide a 2-approximation algorithm which is shown to perform well empirically. Looking ahead, computing distances between labeled trees opens up a variety of new algorithmic directions.Implementation and simulations available at https://github.com/DessimozLab/pylabeledrf.

## Background

Phylogenic trees represent the evolutionary relationship between sets of genetic elements or taxa, where the elements of a set are in one-to-one relationship with the leaves of the corresponding tree [1]. Different phylogenetic inference methods may lead to different trees, and each method, typically exploring a large space of trees, can also result in multiple equally likely solutions for the same dataset. It follows that comparing trees is an essential task for finding out how inferred trees are far from one another, or how an inferred tree is far from a simulated tree or from a gold standard tree for the same datasets.

Designing appropriate tree metrics is a widely explored branch of research. A variety of measures have been designed for different types of trees, rooted or unrooted, some restricted to comparing tree shapes [2], others considering multilabeled trees, i.e. trees with repeated leaf labels [3] and yet others considering information on edge length [4]. In particular, a large number of pairwise measures of similarity or dissimilarity have been developed for comparing two topologies on the same leafset. Among them are the methods based on counting the structural differences between the two trees in terms of path length, bipartitions or quartets for unrooted trees, clades or triplets for rooted trees [5–7], or those based on minimizing a number of rearrangements that disconnect and reconnect subpieces of a tree, such as nearest neighbour interchange (NNI), subtree-pruning-regrafting (SPR) or Tree-Bisection-Reconnection (TBR) moves [8–10]. While the latter methods are NP-hard [11], the former are typically computable in polynomial time. In particular, the Robinson-Foulds (RF) distance, defined in terms of bipartition dissimilarity for unrooted trees, and clade dissimilarity for rooted trees [12], can be computed in linear [13], and even sublinear time [14].

Despite several drawbacks such as lack of robustness (a small change in a tree may cause a disproportional change in the distance), skewed distribution [15–17], and a lack of biological rationale, RF remains the most widely used measure, not only in phylogenetics, but also in other fields such as in linguistics. To increase robustness, improved versions of the RF distance have also been developed [11, 18].

In addition of being efficiently computable, RF has the merit of being a true metric. It was originally defined on unrooted trees, in terms of edit operations on the tree edges: the minimum number of edge contraction and extension needed to transform one tree into the other [19]. Interestingly, the same metric, expressed in terms of node deletion and insertion, has been widely used in the context of data featuring hierarchical dependencies, modeled as trees with labeled nodes. In this case, the standard Tree Edit Distance (TED) is defined in terms of a minimum cost path of node deletion, node insertion and node relabeling (label substitution) transforming one tree to the other, for two trees sharing the same set of node labels (i.e. each label is present exactly once in each tree). While the less constrained version of the problem on unordered labeled trees is NP-complete [20], most variants are solvable in polynomial time [21–23].

Even though this kind of hierarchical node labeling has limited applicability for phylogenetic trees, other types of labeling can be used in the context of genetic data comparison. In the case of gene trees, it is important to identify the evolutionary event (duplication, speciation, transfer, etc) that has led to a given bifurcation. For example, information on duplication and speciation node labeling is provided for the trees of the Ensembl Compara database [24] (reconciled with *TreeBest* [25]). Therefore, being able to compare labeled phylogenies is important in the context of gene tree reconstruction and analysis.

This paper is the first effort towards extending the RF distance to labeled trees involving, in addition to edge contraction and extension (operations that can alternatively be defined as node insertion and deletion), a node substitution or “relabeling” operation. Importantly, our extended RF remains a metric in the mathematical sense.

While the formulation of the RF distance in terms of edit operations is known, the bipartition and clade formulations are often those that are used in the literature. Though similar, the three formulations present some differences depending on whether the trees are rooted or unrooted. We begin by making these differences explicit. We then explore some properties of the extended RF distance in the case of two labels (e.g. speciation and duplication). In particular, we show that, in contrast to the RF distance for unlabeled trees, an optimal edit path for labeled trees may involve contracting good edges, i.e. edges representing common bipartitions of the two compared trees, which makes the extended RF much harder to compute than the basic RF. We then explore various avenues for computing the extended RF. We give an exact algorithm for contracting “mixed subtrees”, i.e. subtrees with alternating labels, and a bounded heuristic for general trees that achieves a factor 2 approximation. In the following section, the heuristic is shown, on simulated datasets, to be efficient, by plotting the number of tree edits against the computed RF distance. Finally, we explore some avenues for improvement. All proofs are given in the Appendix.

## Methods

We first start with notations and concepts, and then describe the Robinson Foulds distance and the extension to labeled trees.

Let *T* be a tree with a node set *V*(*T*) and an edge set *E*(*T*). Given a node *x* of *T*, the *degree of x* is the number of edges incident to *x*. We denote by *L*(*T*)⊆*V*(*T*) the set of *leaves of T*, i.e. the set of nodes of *T* of degree one. A node of *V*(*T*)∖*L*(*T*) is called an *internal node*. A tree with a single internal node is called a *star tree*. An edge connecting two internal nodes is called an *internal edge*; otherwise, it is a *terminal edge*. Moreover, a *rooted tree* admits a single internal node *r*(*T*) considered as the root.

Let *x* and *y* be two nodes of a rooted tree *T*; *y* is an *ancestor* of *x* if *y* is on the path from *x* to the root (possibly *y* itself); *y* is a *descendant* of *x* if *y* is on the path from *x* to a leaf (possibly *y* itself) of *T*. For a rooted tree, we may write (*x*,*y*) for an edge between *x* and *y* where *x* is closer to the root. We say that *y* is a *child* of *x*. If *T* is unrooted, we call the set {*y*:{*x*,*y*}∈*E*(*T*)} the set of children of *x* (this is an unusual definition, but defining a notion of children for both rooted and unrooted trees will be useful later). For a rooted or an unrooted tree *T*, we denote by *Ch*(*x*) the set of children of an internal node *x* of *T*.

A tree *T* representing the evolution of a set ${\mathcal {L}}\/$ of entities (usually taxa or genes) is a tree with a one-to-one mapping between *L*(*T*) and ${\mathcal {L}}\/$. We simply write ${\mathcal {L}}\/ = L(T)$ and say that *T* is a *tree for* ${\mathcal {L}}$. An internal node represents an ancestral event (classically a speciation or a duplication) leading from one to many different entities. Moreover rooting a tree amounts to determining the common ancestor of all entities, i.e. determining the direction of evolution. Accordingly, internal nodes of an evolutionary tree (which are the trees considered in this paper) should be of degree at least 3, except the root which is of degree at least 2. An internal node *x*≠*r*(*T*) of a tree *T* is *binary* if and only if *x* is of degree 3 and *r*(*T*) is *binary* if and only if *r*(*T*) is of degree 2. A tree *T* is said *binary* if and only if all its internal nodes are binary.

A *subtreeS* of *T* is a tree such that *V*(*S*)⊆*V*(*T*),*E*(*S*)⊆*E*(*T*) and any edge of *E*(*S*) connects two nodes of *V*(*S*). A *chain* of *T* is a subtree *C* with a node set *V*(*C*)={*x*_1_,⋯,*x*_*k*_} and an edge set *E*(*C*)={*e*_1_,⋯,*e*_*k*−1_} such that for each 1≤*i*≤*k*,*e*_*i*_ is incident to *x*_*i*_ and *x*_*i*+1_.

If *T* is an unrooted tree, an unrooted version of *T* can just be *T* ignoring the root status of *r*(*T*). To avoid having nodes of degree two, we rather define the *unrooted tree T’ corresponding to T* as the unrooted tree obtained from *T* by adding a dummy leaf *R* and an edge *e*=(*r*(*T*),*R*).

For a rooted tree *T*, we denote by *T*_*x*_ the subtree of *T* rooted at *x*∈*V*(*T*), i.e. the subtree of *T* containing all the descendants of *x*. We call *L*(*T*_*x*_) the *clade of x*. A clade is *non-trivial* if it corresponds to an internal node of *T*. We denote by ${\mathcal {C}}(T)$ the set of non-trivial clades of *T*. It can be seen as a subset of the power set of ${\mathcal {L}}$.

The *bipartition* of an unrooted tree *T* corresponding to an internal edge *e*={*x*,*y*} is the unordered pair of clades *L*(*T*_*x*_) and *L*(*T*_*y*_) where *T*_*x*_ and *T*_*y*_ are the two subtrees rooted respectively at *x* and *y* obtained by removing *e* from *T*. A bipartition is *non-trivial* if it corresponds to an internal edge of *T*, and trivial otherwise. We denote by ${\mathcal {B}}(T)$ the set of non-trivial bipartitions of *T*. Note that bipartitions are sometimes called *splits* in the literature.

### The Robinson-Foulds distance

#### **Definition 1**

(edit operations) Two edit operations on the edges of a tree *T* (rooted or unrooted) are defined as follows:

- Let *e*={*x*,*y*} be an internal edge of *E*(*T*). An *edge contraction* *Cont*(*T*,*e*) is an operation transforming the tree *T* into the tree *T*^′^ obtained from *T* by removing the edge *e* of *T* and identifying *x* and *y*; in other words, *T*^′^ is obtained by adding the edge {*x*,*z*} for each *z*∈*Ch*(*y*)∖{*x*}, and then removing *y* and its incident edges (including {*x*,*y*}).
- Let *x* be a non-binary internal node of *V*(*T*) and $X = \left \{y_{1}, \cdots, y_{t}\right \} \subsetneq Ch(x)$ be a subset of *Ch*(*x*) such that |*X*|≥2. A *node extension* *Ext*(*T*,*x*,*X*) is an operation transforming the tree *T* into the tree *T*^′^ obtained from *T* by removing the edges {*x*,*y*_*i*_}, for 1≤*i*≤*t*, creating a node *y* and a new edge *e*={*x*,*y*} adjacent to *x*, and creating new edges {*y*,*y*_*i*_}, for 1≤*i*≤*t*.

The function *δ*(*T*_1_,*T*_2_) assigning to each pair of rooted or each pair of unrooted trees the length of a minimum sequence of edit operations transforming *T*_1_ into *T*_2_ has been shown to be a metric, called the *Edit distance* or *Robinson-Foulds* distance between *T*_1_ and *T*_2_ [19].

For unrooted trees *T*_1_ and *T*_2_, this distance corresponds to the symmetric difference between the bipartitions of the two trees. More precisely, $\delta \left (T_{1},T_{2}\right) = |{\mathcal {B}}\left (T_{1}\right) \setminus {\mathcal {B}}(T_{2})| + |{\mathcal {B}}(T_{2}) \setminus {\mathcal {B}}(T_{1})|$. In fact, to transform *T*_1_ into *T*_2_, edit operations are needed on *bad edges* representing bipartitions which are not shared by the two trees, i.e. edges of *T*_1_ (respec. *T*_2_) defining bipartitions in *T*_1_ (respec. *T*_2_) which are not in ${\mathcal {B}}(T_{2})$ (respec. in ${\mathcal {B}}(T_{1})$). An edge which is not bad is said to be *good*. Terminal edges are always good.

In the case of rooted trees *T*_1_ and *T*_2_, the Robinson-Foulds distance, that we denote in this case *δ*_*R*_(*T*_1_,*T*_2_), is usually defined in the literature as the symmetric difference between the clades of the two trees. More precisely, for two rooted trees *T*_1_ and $T_{2}, \delta _{R}(T_{1},T_{2}) = |{\mathcal {C}}(T_{1}) \setminus {\mathcal {C}}(T_{2})| + |{\mathcal {C}}(T_{2}) \setminus {\mathcal {C}}(T_{1})|$.

The link between the distance defined in terms of clades (that we write *δ*_*R*_) and the edit distance (that we write *δ*) has been established through the defined relation between the bipartition system (or split system) and the clade system (or cluster system) [26].

Although our extended distance is more likely useful for rooted trees, algorithmic analyses are simpler for unrooted trees, as in this case all internal nodes can be treated in the same way. Here, we make the link between the rooted and unrooted case, and then focus, for the rest of the paper, on unrooted trees.

Let *T*^*r*^ be a rooted version of an unrooted tree *T*, with a binary root. Denote by *e*_1_,*e*_2_ the two edges adjacent to *r*(*T*^*r*^). As *e*_1_ and *e*_2_ define the same bipartition of ${\mathcal {B}}(T)$, these edges are either both good or both bad. These notations are used in the following lemma.

#### **Lemma 1**

(Link between rooted and unrooted trees) Let *T*_1_,*T*_2_ be two rooted trees, and *T*1′, *T*2′ be the corresponding unrooted trees. Then

$${\kern1.7cm}\delta_{R}(T_{1},T_{2}) = \delta(T'_{1},T'_{2}) $$

The edit distance between two trees (rooted or unrooted) can be computed in linear time with the algorithm proposed by Day [13] in 1984. Our goal is to extend this distance to labeled trees.

### Labeled trees

Given a finite set of labels *Λ*, *T is labeled* if and only if each internal node *x* of *T* has a unique label *λ*(*x*)∈*Λ*.

**Fig. 1:**
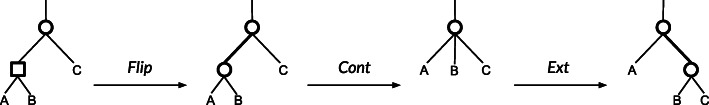
The three edit operations defined for labeled trees. From left to right: Flip, Contraction and Extension

Contraction and extension operations are generalized to labeled trees as follows: The node *y* created from an edge extension *Ext*(*T*,*x*,*X*) is such that *λ*(*y*)=*λ*(*x*); an edge contraction is only defined on edges {*x*,*y*} for which *λ*(*x*)=*λ*(*y*). It follows that a third edit operation should be introduced for labeled trees. Let *x* be a node of a labeled tree *T* with label *λ*=*λ*(*x*). A *node flip* *Flip*(*x*,*λ*^′^) is an operation assigning a new label *λ*^′^ to *x*, i.e. a label *λ*^′^∈*Λ* such that *λ*^′^≠*λ*. Those operations are depicted in Fig. 1.

A node flip is required before contracting a *mixed edge*, i.e. an edge with its two extremities being differently labeled. A tree is said to be a *mixed tree* if all its edges are mixed edges.

Let ${\mathcal {T}}$ be the set of trees on ${\mathcal {L}}$, all trees being of the same type, i.e. all rooted or unrooted, all labeled or unlabeled. The following lemma (holding for all these cases) shows that introducing the flip operation does not prevent *δ* from being a distance.

#### **Lemma 2**

(Edit distance) The function *δ*(*T*_1_,*T*_2_) assigning to each pair $\left (T_{1}, T_{2}\right) \in {\mathcal {T}}^{2}$ the minimum length of a sequence of edit operations transforming *T*_1_ into *T*_2_ defines a distance on ${\mathcal {T}}$.

**Fig. 2:**
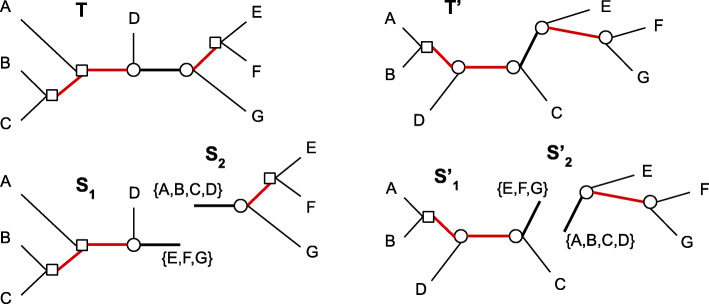
Two unrooted and labeled trees *T* and *T*^′^ on ${\mathcal L} = \{A, B, C, D, E, F, G\}$. The square and circle symbols represent the two possible labels for an internal node. Bad edges are red and good ones are black. {*S*_1_,*S*_2_} are the maximal bad subtrees of *T* and {*S*1′,*S*2′} the corresponding subtrees of *T*^′^

In this paper, *Λ* is restricted to two labels. They are illustrated by a circle and a square in Fig. 2. The two labels can, for example, represent speciation and duplication events. Notice however that labeling is not constrained to be consistent with a species tree [27, 28]. In other words, the intermediate trees in an optimal path transforming a tree to another are not required to be feasible according the speciation/duplication labeling. Algorithmic analyses are made independently of the nature of the two node labels. However, for notation purpose, we write *Λ*={*Spe*,*Dup*}.

## Results

Consider ${\mathcal {T}}$ as the set of unrooted and labeled trees on ${\mathcal L}$. The goal is to compute the edit distance *δ*(*T*,*T*^′^) for any pair *T*,*T*^′^ of trees of ${\mathcal {T}}$, that is the number of operations in an *optimal sequence*, i.e a sequence of edit operations of minimum length transforming *T* into *T*^′^.

Note that although we focus on unrooted trees, our results can then be easily extrapolated to rooted trees using Lemma ??,

### Reduction to maximal bad subtrees

Let *S* be a subtree of *T*. Let {*e*_*i*_={*x*_*i*_,*y*_*i*_}, for 1≤*i*≤*k*} be the set of terminal edges of *S*, with each *y*_*i*_ being a leaf of *S*, and {*X*_*i*_,*Y*_*i*_} being the bipartition corresponding to *e*_*i*_. Each leaf *y*_*i*_ of *S* is said to be *mapped* to *Y*_*i*_. Notice that $\cup _{1 \leq i \leq k}Y_{i} = {\mathcal L}$.

We say that *S* is a *bad subtree of T* if and only if *S* contains only bad edges, except the terminal edges of *S* which are all good edges of *T*. In other words, *S* is maximal in the sense that no more bad internal edges can be added into it. Intuitively, *S* can be obtained by taking a subtree with only bad edges, and adding edges adjacent to bad edges of *S* iteratively until the process stops. As a result, every terminal edge *e*_*i*_ of *S* will be good, i.e. there is an edge $e^{\prime }_{i} = \left \{x'_{i}, y'_{i}\right \}$ in *T*^′^ corresponding to *e*_*i*_={*x*_*i*_,*y*_*i*_}, that determine the same bipartition {*X*_*i*_,*Y*_*i*_}. Note that a maximal bad subtree may contain no bad edge at all (i.e. it is a star tree centered on good edges).

#### **Lemma 3**

(Pairs of maximal bad subtrees) Let *S* be a maximal bad subtree of *T* with the set {*e*_*i*_}_1≤*i*≤*k*_ of terminal edges, and let {*ei*′}_1≤*i*≤*k*_ be the corresponding set of edges in *T*^′^. Then the subtree *S*^′^ of *T*^′^, containing all $e^{\prime }_{i}$ edges as terminal edges, is unique. Moreover, it is a maximal bad subtree of *T*^′^.

Let {*S*_1_,*S*_2_,⋯,*S*_*k*_} be the set of maximal bad subtrees of *T* and {*S*1′,*S*2′,⋯,*Sk*′} be the corresponding subtrees of *T*^′^ (see Fig. 2 for an example). For 1≤*i*≤*m*, let ${\mathcal {P}}_{i}$ be an optimal sequence transforming *S*_*i*_ into $S^{\prime }_{i}$. Then the sequence ${\mathcal {P}}$ obtained by performing consecutively ${\mathcal {P}}_{1}, {\mathcal {P}}_{2}, \cdots, {\mathcal {P}}_{m}$ transforms *T* into *T*^′^.

**Fig. 3:**
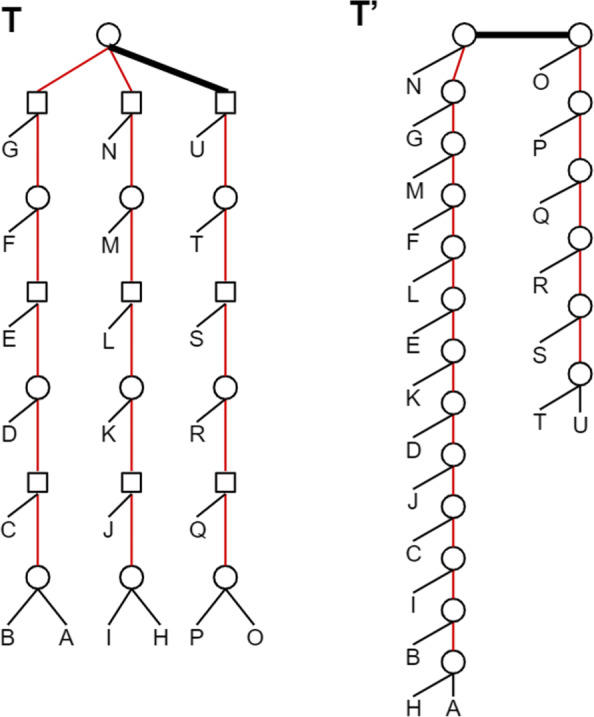
Example where the minimal edit path requires contracting a good edge: if we contract the internal good edge of *T* (the bold one), then the 3 subtrees of *T* can be handled together, requiring 6 node flips and 18 edge contractions to reduce *T* into a star tree, and then 18 edge extensions to reach *T*^′^, leading to 42 operations in total. By contrast, if we do not contract the good edge of *T*, then the two subtrees of *T* separated by this edge should be handled separately, requiring 9 flips, 17 edge contractions and 17 edge extensions to reach *T*^′^, leading to 43 operations in total. The first scenario is the better one

Although the traditional RF distance can be deduced from the above observation, in our case such a sequence is not necessarily optimal. In fact, in contrast with unlabeled trees, optimal sequences for labeled trees may involve contracting good edges, as illustrated in Fig. 3.

### Reduction to mixed bad subtrees

In the next section, we will describe an exact algorithm for optimally contracting a mixed tree. Before reaching this step, the question is how to obtain such a tree. The next lemma shows that non-mixed bad edges can be contracted first. The idea of the proof is that any optimal solution must eventually contract a non-mixed bad edge {*x*,*y*}. We can thus contract {*x*,*y*} first into a single node *z*, and “reproduce” all the events of the optimal solution by treating *z* as either *x* or *y*.

#### **Lemma 4**

(Contract non-mixed bad edges) Let *e* be any non-mixed bad edge of *T*, and let *T*_*c*_ be the tree obtained from *T* by contracting *e*. Then *δ*(*T*_*c*_,*T*^′^)=*δ*(*T*,*T*^′^)−1.

According to this lemma, we can safely start by contracting all non-mixed bad edges of *T* and *T*^′^ first, since there is always an optimal sequence of edit operations that also does this. The resulting trees *T*_*c*_ and $T^{\prime }_{c}$ can then be subdivided into pairs of maximal bad subtrees, all such bad subtrees being mixed subtrees.

### Algorithms

We first consider a general framework which entails performing all required edge contractions first, and then all node extensions.


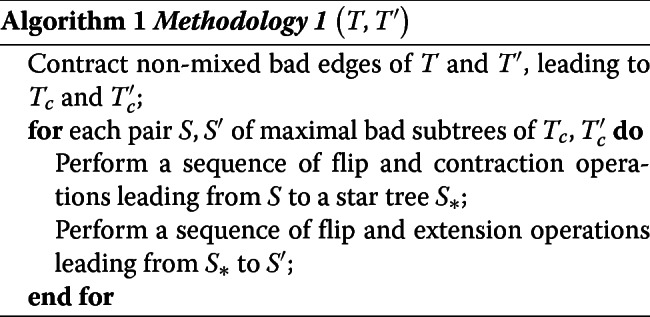


This general framework leads to the following upper bound for *δ*(*T*,*T*^′^).

#### **Lemma 5**

(Upper bound *δ*) Let *T* and *T*^′^ be two unrooted and labeled trees with *n* internal nodes each and let *e* (resp. *e*^′^) be the number of internal bad edges of *T* (resp. *T*^′^). Then *δ*(*T*,*T*^′^)≤*e*+*e*^′^+*n*.

**Fig. 4:**
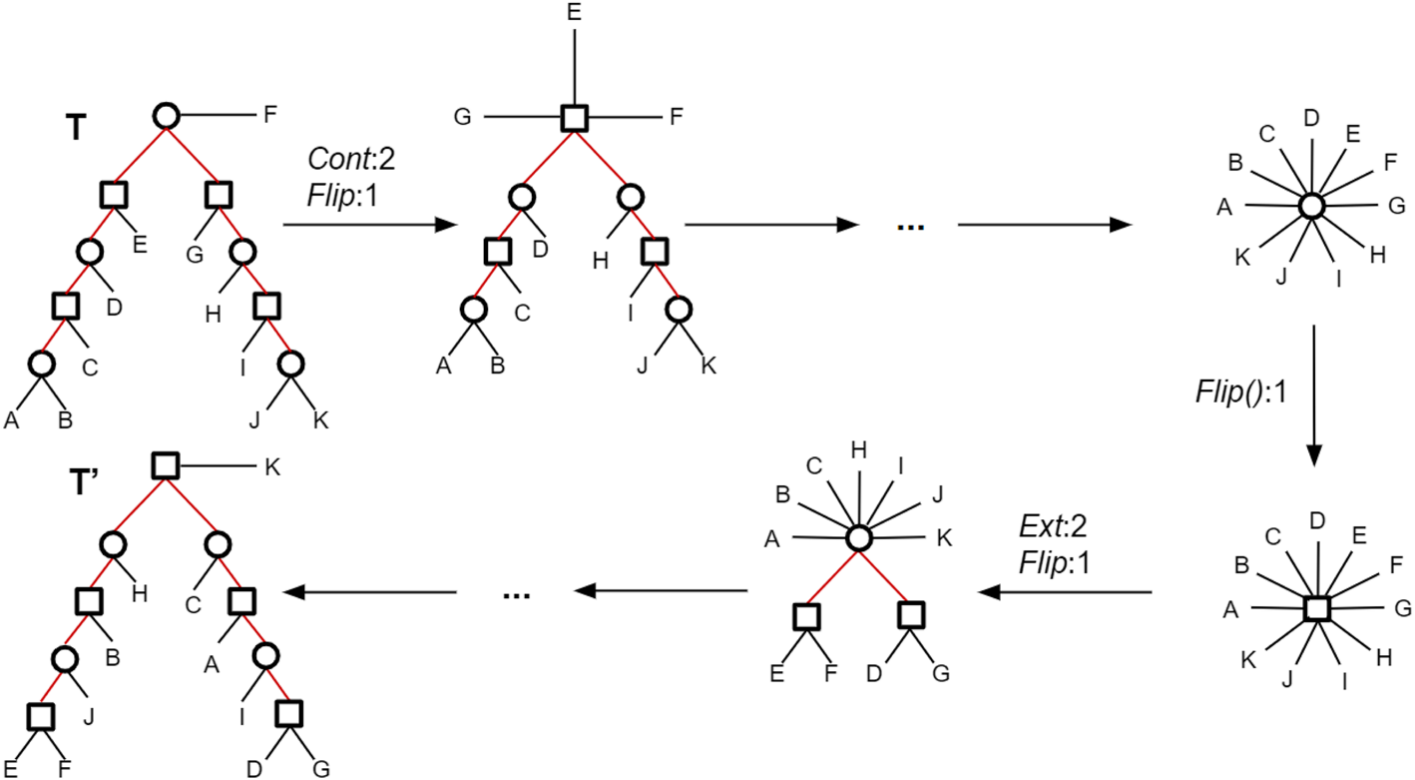
A pair of unrooted mixed trees (*T*,*T*^′^), both with eight internal edges and nine internal nodes. All their internal edges are bad edges (red edges). Here *δ*(*T*,*T*^′^)=25=2·8+9=2·*e*+*n*

Notice that if both *T* and *T*^′^ are binary, then *e*=*e*^′^. Moreover, in this case 2*e*+*n* is a tight bound as it can be reached in some cases (see an example in Fig. 4).

The first step of Methodology 1 leads to a star tree *T*_∗_. Instead of then extending nodes to reach *T*^′^, a symmetric way would be to transform *T*^′^ into a star tree $T^{\prime }_{*}$. The difference between *T*_∗_ and $T^{\prime }_{*}$ may be in the label of the single node of each of these trees, which would then need an additional flip operation to reconstruct a corresponding path from *T* to *T*^′^. This second methodology is given below, where *Contract-Tree* (*T*,*T*_∗_) takes as input a tree *T* and returns a sequence of operations *contracting a tree T*, i.e. transforming *T* into a star tree, and the star tree *T*_∗_ resulting from this optimal contraction.

Methodology 2 is clearly simpler to handle and will be explored in the next section. The next lemma shows that it may overestimate an optimal sequence returned by Methodology 1 by at most one operation for each pair of maximal bad subtrees.


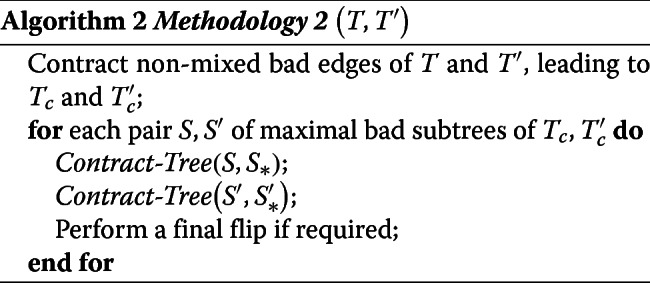


#### **Lemma 6**

(Compare Meth.1 and Meth.2) Let *S* and *S*^′^ be a pair of maximal bad subtrees of *T*_*c*_ and $T^{\prime }_{c}$, obtained similarly by Methodology 1 and Methodology 2. Let *M*_1_(*S*,*S*^′^) (respec. *M*_2_(*S*,*S*^′^)) be the number of operations performed by the for loop of Methodology 1 (respec. Methodology 2). Moreover, let *S*_∗_ (respec. $S^{\prime }_{*}$) be the star tree returned by *Contract-Tree* on *S* (respec. on *S*^′^).

1. If *S*_∗_=*S*∗′ (same node label), then *M*_2_(*S*,*S*^′^)=*M*_1_(*S*,*S*^′^);
2. Otherwise, *M*_1_(*S*,*S*^′^)≤*M*_2_(*S*,*S*^′^)≤*M*_1_(*S*,*S*^′^)+1

#### An optimal algorithm for contracting a tree

The remaining problem is the one of finding an optimal sequence of contraction and flip operations contracting a mixed tree *T*. For any such sequence, the number of contraction operations is just the number of internal edges of *T*. Therefore, the problem reduces to finding the minimum number of flip operations *ϕ*(*T*) in such an optimal sequence. Notice that the problem does not reduce to performing the minimum number of flips leading to the same label for all nodes, which would just be *min*{*nb*_*spe*_,*nb*_*dup*_} with *nb*_*spe*_ (respec. *nb*_*dup*_) being the number of *Spe* (respec. *Dup*) nodes of *T*. For example, for the tree *T* of Fig. 3, *min*{*nb*_*spe*_,*nb*_*dup*_}=9. However, proceeding by an alternating sequence of flip and contraction operations (the top node flipped to *Dup*, then the three top edges contracted, then the next top node flipped to a *Spe* node, then the three top edges contracted, etc.) leads to a total of 6 flips rather than 9.

We will proceed iteratively by starting a sequence of contraction operations from the center of a tree *T*, i.e. the midpoint of the longest mixed chain of *T*. The *diameter*, denoted *diam*(*T*), of a tree *T* is the length of its longest chain (determined in terms of the number of edges). Note that any longest chain in a tree has two leaves at its extremities, as otherwise we could extend the chain. Assume that *T* has at least two terminal edges, so that *diam*(*T*)≥2. We show that *ϕ*(*T*) is equal to ⌈*diam*(*T*)/2⌉−1. For a node *v*, let *ecc*_*T*_(*v*) denote the maximum distance from *v* to a leaf of *T* (this is known as the *eccentricity* of *v*).^1^

##### **Lemma 7**

(Optimal path contracting a mixed tree) The minimum number of flips in an optimal sequence of operations transforming a mixed tree *T* into a star tree is ⌈*diam*(*T*)/2⌉−1.


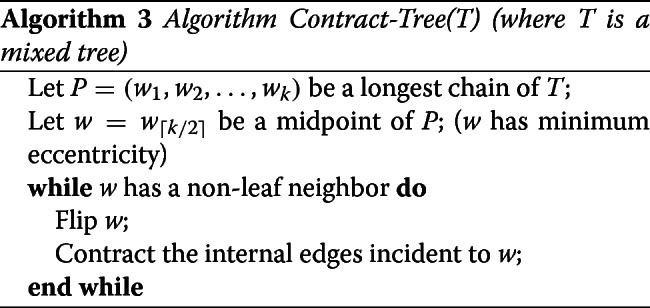


Lemma ?? immediately lead to Algorithm *Contract-Tree*. The fact that the algorithm contracts *T* into a star tree using *ϕ*(*T*) flips follows from the proof of Lemma ??.

##### **Theorem 1**

For *T* being a mixed tree, Algorithm Contract-Tree returns the length of an optimal sequence of operations contracting *T*.

One should note that if *T* has even diameter, then there are two possible midpoints, i.e. two nodes with minimum eccentricity. This means that it is possible to choose the label of the internal node of the resulting star tree. This guarantees that when contracting a pair of bad subtrees *T* and *T*^′^, we can always avoid a final flip by choosing the appropriate final label if either *T* or *T*^′^ has even diameter. We cannot guarantee that this final flip is avoidable if both subtrees have odd diameter.

We now show that Methodology 2 has a guaranteed approximation ratio of 2 when using Algorithm *Contract-Tree* as a subroutine. The idea behind the approximation is to show that any optimal solution must contract all the bad edges and perform at least one flip or good edge contraction per bad subtree. Our algorithm only contracts bad edges, and we can show that the number of flips performed is at most the number of bad edges plus twice the number of bad subtrees.

##### **Theorem 2**

(Upper bound Meth.2) Let *d* be the number of operations performed by Methodology 2 when tree contractions are done by Algorithm *Contract-Tree*. Then *d*≤2*δ*(*T*,*T*^′^).

### Experimental results

We implemented a heuristic following Methodology 2, using the *Contract-Tree* algorithm. To test it on simulated data, we retrieved the TP53 gene family from Ensembl release 96 (542 genes), including the speciation and duplication labels, and introduced an increasing number of random edit operations, on 30 replicates. A random edit was introduced as follows: with probability 0.3, the label of one random internal node was flipped; the rest of the probability mass function was evenly distributed among all internal edges connecting nodes of the same type (which could be potentially contracted) and all nodes of degree >3 (in which a new edge could potentially be expanded).

**Fig. 5:**
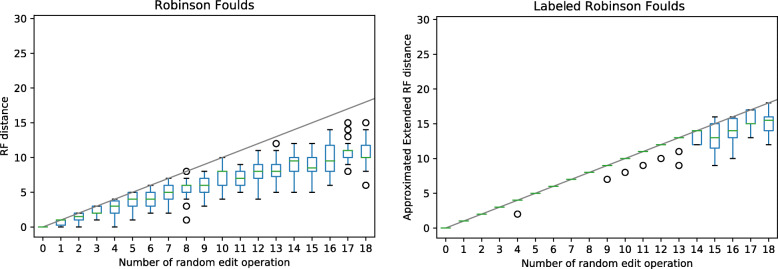
Empirical comparison of the distance inferred for an increasing number of random edit operations (contraction, extensions, and flips), using the classical Robinson-Foulds distance (left) and *Contract-Tree* algorithm (right). Because the former ignores node labels, it grossly underestimates the actual number of edits. Our algorithm tracks more closely the actual number of edits

After each edit, we computed the classical RF distance and its extension to labeled trees using our heuristic (Fig. 5). Because it accounts for labels, the latter tracked more closely the true number of edits. At the same time, the estimated distances were never higher than the actual number of edits, which suggests that the heuristic can identify a minimum edit path when the total number of edit operations is relatively low. The implementation, including the function to mutate labeled trees, is available as an open source Python library (PyPI package pylabeledrf, also available at https://github.com/DessimozLab/pylabeledrf).

## Discussion

In this paper, we have considered what we thought was the simplest and most natural extension of the Robinson-Foulds distance to labeled trees. Although its theoretical complexity is unknown and remains an open problem, this extension appears to be much harder to compute than the classical RF distance for unlabeled trees.

**Fig. 6:**
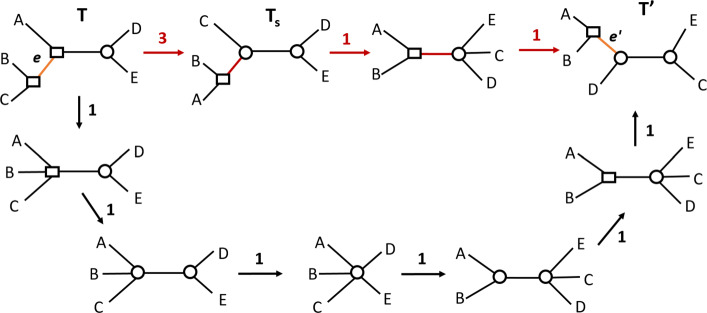
An optimal path from *T* to *T*^′^ following *Methodology 1* is depicted by black arrows and involves 6 operations. It is not optimal as another path, depicted by red arrows, involves only five operations. The path of length 3 from *T* to *T*_*s*_ acts on the safe edge, represented in orange. This path involves an edge contraction, an edge extension and a flip, leading to the good edge (red edge) in *T*_*s*_

Despite the optimality of Algorithm *Contract-Tree* for contracting a mixed tree, neither *Methodology 1*, nor *Methodology 2* are guaranteed to lead to an optimal solution. This is due to two main reasons. The first one is that, as shown in Fig. 3, an optimal path contracting a tree *T* may require contracting good edges, i.e. edges common to both trees, which is not the case for unlabeled trees. The second reason is that an optimal path from a tree *T* to a tree *T*^′^ may not be one with all edge contraction events preceding all edge extension. An example, given in Fig. 6, shows that it may be better to convert a given bad edge into a good edge rather than contracting all bad edges. It can be observed from this example that going from *T* to *T*^′^ following the red path entails performing a nearest-neighbour interchange (NNI) operation on the edge *e* of *T*. A future direction for improving the algorithm will be to consider such “safe” edges, i.e. edges admitting an NNI leading to a bipartition of the target tree.

Still, we have implemented a heuristic which constitutes a better baseline solution to quantifying differences between labeled tree topologies than the conventional RF measure, which is blind to labels. For instance, this implementation could be useful in the context of orthology benchmarking, to compare inferred labeled trees with reference curated ones [29].

## Conclusion

Looking ahead, we envision several potential future directions. We see potential in identifying the good edges that should be contracted and characterizing classes of trees that may be resolved optimally. In particular, it would be interesting to restrict the study to the class of labeled trees consistent with a species tree (which is not the case of the trees of Fig. 3).

Another direction would be to consider an alternative extension of the RF distance. In this paper, edge contraction and edge extension, the two edit operations defining the classical RF, were re-defined in the context of labeled nodes, by constraining them to occur on edges with the same labels on their extremities. Another direction would be to consider edit operations on nodes, as for the Tree Edit Distance (TED) for hierarchical trees, i.e. node deletion, insertion and relabeling. In addition to the theoretical complexity and computational efficiency, it would be important to evaluate the robustness of these two RF extensions with respect to small changes in the topology or tree labeling. Although we do not expect robustness to be much better than the classical RF, knowing which extension is better can orient the study towards future improvements. Finally another direction would be to extend the study to an arbitrary set of possible labels.

More generally, we think that computing the distance between labeled trees conceals many new problems and opens a variety of new algorithmic directions.

## Appendix

### Proof of Lemma ?? (Link between rooted and unrooted trees)

Let *T*_1_ and *T*_2_ be two rooted trees and $T^{\prime }_{1}$ and $T^{\prime }_{2}$ be the corresponding unrooted trees, i.e. *V*(*T*1′)=*V*(*T*_1_)∪{*R*},*V*(*T*2′)=*V*(*T*_2_)∪{*R*},*E*(*T*1′)=*E*(*T*)∪{(*r*(*T*_1_),*R*)} and *E*(*T*2′)=*E*(*T*)∪{(*r*(*T*_2_),*R*)}.

We first show that any bad bipartition of $T^{\prime }_{1}$, i.e. any bad edge of $T^{\prime }_{1}$, corresponds to a bad clade of *T*_1_ (a clade which is not present in *T*_2_). Let $e^{\prime }_{1}$ be a bad edge of $T^{\prime }_{1}$. Then $e^{\prime }_{1}$ should be a non-terminal edge of $T^{\prime }_{1}$, thus different from (*r*(*T*_1_),*R*)), and therefore it has a corresponding edge *e*_1_=(*x*_1_,*y*_1_) in *T*_1_. Then, for one of the two nodes adjacent to $e^{\prime }_{1}$ that we denote *y*1′, we have $L(T'_{1y'_{1}}) = L(T_{1y_{1}})=C$. If *e*1′ is a bad edge of $T^{\prime }_{1}$, then *C* should be a bad clade of *T*_1_ not present in *T*_2_. This is because otherwise *C* would be a non-trivial clade of *T*_2_ rooted at an internal node *y*_2_ adjacent to an edge *e*_2_=(*x*_2_,*y*_2_) and thus also equal to $L(T'_{2y'_{2}})$ for a given edge *e*2′=(*x*2′,*y*2′). This contradicts the fact that $e^{\prime }_{1}$ is a bad edge. Therefore, each bad bipartition of $T^{\prime }_{1}$ corresponds to a bad clade of *T*_1_. Moreover, two disjoint bad bipartitions of $T^{\prime }_{1}$ correspond to two different bad edges of $T^{\prime }_{1}$, with the corresponding edges of *T*_1_ associated to two disjoint clades. Thus we have $|\mathcal {B}(T'_{1})| \leq |\mathcal {C}(T_{1})|$.

Conversely, a bad clade *C* of *T*_1_ corresponds to an internal node *y*_1_ of *T*_1_. Let *e*_1_=(*x*_1_,*y*_1_) in *T*_1_, where *x*_1_ is the parent of *y*_1_. Then the corresponding edge $e^{\prime }_{1}$ in $T^{\prime }_{1}$ is a bad edge. Moreover, two disjoint clades of *T*_1_ correspond to two disjoint edges of *T*1′. It follows that $|\mathcal {C}(T_{1})| \leq |\mathcal {B}(T'_{1})|$. Combining this result with the result above, we deduce that $|\mathcal {C}(T_{1})| = |\mathcal {B}(T'_{1})|$. As *T*_2_ and $T^{\prime }_{2}$ can be considered similarly, the result follows.

### Proof of Lemma ?? (Edit distance):

The non-negative and identity conditions are obvious. For the symmetric condition, notice that we can reverse every edit operation in an optimal sequence from *T*_1_ to *T*_2_ to obtain a sequence from *T*_2_ to *T*_1_ with the same number of events, and vice-versa (extensions and contractions are inverses of each other, and any flip can be reversed by a flip). We thus have *δ*(*T*_2_,*T*_1_)≤*δ*(*T*_1_,*T*_2_) and *δ*(*T*_1_,*T*_2_)≤*δ*(*T*_2_,*T*_1_), and equality follows.

Finally, we prove the triangular inequality condition: for 3 trees *T*_1_,*T*_2_ and *T*_3_, to transform *T*_1_ into *T*_2_, we may take any edit sequence from *T*_1_ to *T*_3_, followed by any edit sequence from *T*_3_ to *T*_2_. It follows that *δ*(*T*_1_,*T*_2_)≤*δ*(*T*_1_,*T*_3_)+*δ*(*T*_3_,*T*_2_).

### Proof of Lemma ?? (Pairs of maximal bad subtrees):

As ∪_*i*_*Y*_*i*_=Ł, $\phantom {\dot {i}\!}\{e'_{i}\}_{1 \leq i \leq k}$ are the only terminal edges of any subtree *S*^′^ of *T*^′^ containing the set $\phantom {\dot {i}\!}\{e'_{i}\}_{1 \leq i \leq k}$ as terminal edges. As *T*^′^ is a tree, for any 1≤*i*≠*j*≤*k*, there is only one possible path from $x^{\prime }_{i}$ to $x^{\prime }_{j}$. Uniqueness follows.

Suppose that such a subtree *S*^′^ is not a bad subtree. Then it contains an internal good edge *e*^′^=(*x*^′^,*y*^′^). In other words, there is a non-trivial bipartition of {*Y*_*i*_}_1≤*i*≤*k*_ which is also a bipartition in *S*. This contradicts the fact that *S* is a bad subtree of *T*. Finally, as all terminal edges of *S*^′^ are good edges of *T*^′^, it follows that *S*^′^ is a maximal bad subtree of *T*^′^.

### Proof of Lemma ?? (Contract non-mixed bad edges):

We first introduce a definition that will be of use later in the proof. For two rooted trees *S*_1_ and *S*_2_, define the *union* of *S*_1_ and *S*_2_ as the tree obtained by identifying their roots, i.e. by removing the root of *S*_2_ and making all its children now children of the root of *S*_1_.

Let *e*={*u*,*v*} be a non-mixed bad edge and assume, without loss of generality, that both *u* and *v* have the label *Spe* (recall that *Λ*={*Spe*,*Dup*}). Notice that any sequence of operations turning *T* into *T*^′^, at some point, must contract the {*u*,*v*} edge, as otherwise, the (bad) bipartition corresponding to {*u*,*v*} would remain in the transformed tree and we would not obtain *T*^′^ (noting that extensions cannot remove bipartitions). We now prove the Lemma by induction over *δ*(*T*,*T*^′^). As a base case, suppose that *δ*(*T*,*T*^′^)=1. Then {*u*,*v*} must be the only bad edge of *T* and the single operation is to contract it, proving the base case.

Now assume that for any tree $\tilde {T}$ satisfying $\delta (\tilde {T}, T') < \delta (T, T')$, contracting any non-mixed bad edge of $\tilde {T}$ reduces its distance to *T*^′^ by 1. Let *Q*=(*q*_1_,…,*q*_*l*_) be an optimal sequence of operations transforming *T* into *T*^′^ (here each *q*_*i*_ denotes either a contraction, extension or flip). Let *q*_*j*_ be the event that contracts {*u*,*v*}. If *q*_1_=*q*_*j*_, then we are done, so assume otherwise. We make the assumption that whenever there is a contraction involving *u* prior to *q*_*j*_, the contracted node is still called *u*. Furthermore, we assume that if an extension prior to *q*_*j*_ splits the neighbors of *u*, the node *v* is still a neighbor of *u* after the operation. All the same assumptions hold for *v*. This just changes the names we give to nodes and does not alter the scenario, but observe that this means that {*u*,*v*} is in every tree obtained before the first *j* operations.

For each *i*∈{1,…,*l*}, let *T*_*i*_ be the tree obtained after applying *q*_1_,…,*q*_*i*_ on *T*, and define *T*_0_=*T*. Furthermore, for *i*∈{0,1,…,*j*−1}, denote by $T^{u}_{i}$ and $T^{v}_{i}$ the two trees obtained from *T*_*i*_ by removing the edge {*u*,*v*}, where *u* is in $T^{u}_{i}$ and *v* is in $T^{v}_{i}$. Define $T^u = T^{u}_{0}$ and $T^v = T^{v}_{0}$. We will assign *u* and *v* as the respective roots of each $T^{u}_{i}$ and $T^{v}_{i}$. Notice that for each *i*∈{1,…,*j*−1}, *q*_*i*_ only modifies either the subtree $T^{u}_{i-1}$ or $T^{v}_{i-1}$. Therefore, if events *q*_*i*_ and *q*_*i*+1_ modify $T^{u}_{i-1}$ and $T^{v}_{i}$, respectively, we could apply *q*_*i*+1_ before *q*_*i*_ and *T*_*i*+1_ would still be the same tree. This lets us assume that we may reorder events such that all events affecting *T*^*u*^ (prior to *q*_*j*_) occur before those affecting *T*^*v*^. That is, there is some *h* such that *q*_1_,…,*q*_*h*_ only affects the *T*^*u*^ subtree, *q*_*h*+1_,…,*q*_*j*−1_ only affects the *T*^*v*^ subtree, so that $T^{u}_h = T^{u}_{h+1} = \ldots = T^{u}_{j-1}$ and $T^v = T^{v}_1 = \ldots = T^{v}_{h}$.

Suppose first that *u* is labeled *Spe* in *T*_*h*_, and thus also in *T*_*j*−1_. Then *v* is also labeled *Spe* in *T*_*j*−1_ (and also in *T*_*h*_ since *v* was untouched until *q*_*h*+1_). Let $\hat {T}$ be the tree obtained after contracting {*u*,*v*} in *T*, and let *z* be the resulting node. Observe that if we interpret *z* as *u*, then we may apply the events *q*_1_,…,*q*_*h*_ on $\hat {T}$, since these events only affected the *T*^*u*^ subtrees. To be formal, we “reproduce” *q*_1_ through *q*_*h*_ on $\hat {T}$ by applying the events *Q*^′^=(*q*1′,…,*qh*′) on $\hat {T}$, defining $\hat {T}_{i}$ as the tree obtained after the *i*-th event of *Q*^′^, where each $q^{\prime }_{i}$ in *Q*^′^ is defined as follows:

- if *q*_*i*_ contracts {*x*,*y*} in *T*_*i*−1_, then $q^{\prime }_{i}$ contracts {*x*,*y*} in $\hat {T}_{i-1}$ if *x*,*y*≠*u*, otherwise if, say, *x*=*u*, then $q^{\prime }_{i}$ contracts {*z*,*y*} (and calls the resulting node *z*);
- if *q*_*i*_ flips *x* in *T*_*i*−1_, then $q^{\prime }_{i}$ flips *x* in $\hat {T}_{i-1}$ if *x*≠*u*, or flips *z* otherwise;
- if *q*_*i*_ is an extension and splits the neighborhood of *x*, then $q^{\prime }_{i}$ does the same if *x*≠*u* (replacing *u* by *z* if needed). If *x*=*u*, then let *X* be the set of neighbors of *v* in *T*_*i*−1_, excluding *u*. If *Ch*(*u*) is split into *A* and *B* by *q*_*i*_, where *v*∈*B*, then $q^{\prime }_{i}$ splits the neighbors *A*∪*B*∪*X* of *z* into *A* and *B*∪*X* (and *z* is the neighbor of *B*∪*X* and the newly created node).

One can verify the following that the following invariant holds on each $\hat {T_i}, i \in \{1, \ldots, h\}$: if we take *T*_*i*_ and contract the edge {*u*,*v*}, ignoring the labels and keeping the label of *u*, then we obtain $\hat {T}_{i}$ (the invariant is also true for *T* and $\hat {T}$).

The resulting tree $\hat {T}_{h}$ obtained from applying *q*1′,…,*qh*′ on $\hat {T}$ will therefore contain *z* as a *Spe* node, and will be the union of $T^{u}_{h}$ and $T^{v}_{0}$. From this point, in a similar fashion, we may interpret *z* as *v* and apply *q*_*h*+1_,…,*q*_*j*−1_ on $\hat {T}_{h}$, resulting a tree that is the union of $T^{u}_h = T^{u}_{j-1}$ and $T^{v}_{j-1}$. The corresponding events are the same as above, we omit the formal details. Since *T*_*j*_ is obtained from *T*_*j*−1_ by contracting {*u*,*v*}, this means that $\hat {T}_{j-1} = T_{j}$, which we have attained with *j* events but contracting {*u*,*v*} first, which proves this case.

Suppose instead that *u* is labeled *Dup* in *T*_*h*_. Then *v* is a *Dup* node in *T*_*j*−1_. We may further assume that *v* is a *Spe* node in *T*_*h*+1_,…,*T*_*j*−2_, since whenever we flip *v* into a *Dup*, we may assume by induction that {*u*,*v*} gets contracted. Therefore, *q*_*j*−1_ flips *v* from *Spe* to *Dup*, and for the first time. We may then do the following: first apply the events *q*_*h*+1_,…,*q*_*j*−2_ on $\hat {T}$, interpreting *z* as *v*. The resulting tree $\hat {T}'$ contains *z* as a *Spe* node, and is the union of $T^{v}_{j-2}$ and $T^{u}_{0}$. We may now apply *q*_1_,…,*q*_*h*_ on $\hat {T}'$ by interpreting *u* as *z*, resulting in a tree $\hat {T}^{\prime \prime }$ that contains *z* as a *Dup* node and is the union of $T^{u}_{h} = T^{u}_{j-1}$ and $T^{v}_{j - 1}$. We have thus attained *T*_*j*_, but this time without the *q*_*j*−1_ flip on *v*, contradicting the optimality of *Q*. This concludes the proof.

### Proof of Lemma ?? (Upper bound *δ*):

Methodology 1 performs *e* contractions and *e*^′^ extensions. As for the number of flips, we have to flip at most all the nodes belonging to the smallest label group, which means at most half the nodes in each tree, and thus at most *n* flips in total.

### Proof of Lemma ?? (Compare Meth.1 and Meth.2):

**Fig. 7:**
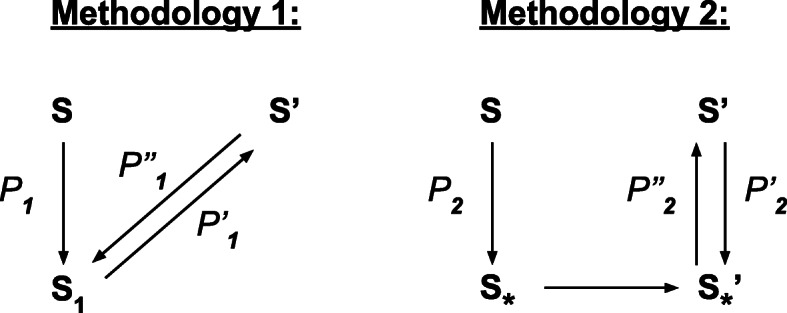
Notations for the Proof of Lemma ??

We denote by *Cont*(*T*) the minimum length of a sequence of operations contracting *T*, and by *l*(*¶*) the length of a sequence *¶* of edit operations (Fig. 7).

Let *¶*_2_ be an optimal sequence contracting *S* to *S*_∗_ and *¶*2′ be an optimal sequence contracting *S*^′^ to *S*∗′. As each operation is reversible, *¶*2′ leads to a corresponding sequence *¶*2′′ of the same length between *S*∗′ and *S*^′^. Thus, *¶*_2_, concatenated with a possible flip operation transforming *S*_∗_ to $S^{\prime }_{*}$, concatenated with *¶*2′′ is a sequence from *S* to *S*^′^ following Methodology 1, and thus *M*_1_(*S*,*S*^′^)≤*M*_2_(*S*,*S*^′^) (R1).

Conversely, let *¶* be an optimal sequence following Methodology 1. Then this sequence can be subdivided into a sequence *¶*_1_ from *S* to a star tree *S*_1_, and *¶*1′ from *S*_1_ to *S*^′^. As each operation is reversible, *¶*1′ leads to a corresponding sequence *¶*1′′ of the same length between *S*^′^ and *S*_1_. In other words, *M*_1_(*S*,*S*^′^)=*l*(*¶*_1_)+*l*(*¶*1′)=*l*(*¶*_1_)+*l*(*¶*1′′)≥*Cont*(*S*)+*Cont*(*S*^′^).

1. If *S*_∗_=*S*∗′, then *M*_2_(*S*,*S*^′^)=*Cont*(*S*)+*Cont*(*S*^′^) and thus *M*_1_(*S*,*S*^′^)≥*M*_2_(*S*,*S*^′^), and the result follows from (R1).
2. Otherwise, *S*_∗_ and *S*∗′ are different and *M*_2_(*S*,*S*^′^)=*Cont*(*S*)+*Cont*(*S*^′^)+1. Thus *M*_1_(*S*,*S*^′^)≥*Cont*(*S*)+*Cont*(*S*^′^)=*M*_2_(*S*,*S*^′^)−1, and thus *M*_2_(*S*,*S*^′^)≤*M*_1_(*S*,*S*^′^)+1.

### Proof of Lemma ?? (Optimal path contracting a mixed tree):

We first show that at least ⌈*diam*(*T*)/2⌉−1 flips are needed, by induction over the diameter of *T*. When *diam*(*T*)=2, *T* is a star tree and 0=*diam*(*T*)/2−1 flips are needed. For the induction step, we assume that any tree *T*^′^ with *diam*(*T*^′^)<*diam*(*T*) requires at least ⌈*diam*(*T*^′^)/2⌉−1 flips. Take any optimal sequence of events *S*, and observe that in *S*, when we flip a node *v* of *T*, by Lemma ?? we may assume that *S* contracts all the incident edges to *v* until we obtain another mixed tree. Let *T*_1_,*T*_2_,…,*T*_*k*_ be the sequence of mixed trees encountered when applying *S*, i.e. each *T*_*i*_ is obtained after flipping a node and contracting its incident edges. Define *T*_0_=*T*. Let *i* be the smallest index such that *diam*(*T*_*i*_)<*diam*(*T*). Then in *T*_*i*−1_, there was a longest chain *P*=(*u*_1_,…,*u*_*l*_) of length *diam*(*T*). The flip-and-contract operations from *T*_*i*−1_ to *T*_*i*_ can reduce the length of *P* by at most 2 since we flip one node and only its incident edges, of which there are at most two on *P*. Hence *diam*(*T*_*i*_)≥*diam*(*T*)−2. We deduce by induction that the number of required flips is at least 1+⌈(*diam*(*T*)−2)/2⌉−1=⌈*diam*(*T*)/2⌉−1.

We now turn to the converse bound *ϕ*(*T*)≤⌈*diam*(*T*)/2⌉−1. Fix any node *v* of *T*, and suppose that we run the following procedure: as long as *T* is not a star tree, flip *v* and contract its incident internal edges. Since each flip-and-contraction iteration reduces the length from *v* to any leaf by 1 (except its neighbors), *ecc*_*T*_(*v*) is reduced by 1 each round. We stop when *ecc*_*T*_(*v*)=1, in which case only terminal edges remain, and in the end, this means that *ecc*_*T*_(*v*)−1 flips are needed.

To see why this proves our bound, we show that there always exists a node with eccentricity ⌈*diam*(*T*)/2⌉. Consider a longest chain *P* of *T* with nodes *w*_1_,…,*w*_*k*_. Observe that *diam*(*T*)=*k*−1 (recall that distances are counted in terms of edges). Consider a midpoint node *w*:=*w*_⌈*k*/2⌉_ on *P*. We claim that *ecc*_*T*_(*w*)=⌈*diam*(*T*)/2⌉. It is easy to check that *w* has distance at most ⌈*diam*(*T*)/2⌉ and at least ⌊*diam*(*T*)/2⌋ to the leaves *w*_1_ and *w*_*k*_ on *P*. Assume for contradiction that *w* is at distance at least ⌈*diam*(*T*)/2⌉+1 from some leaf *l* of *T* not in *P*. Then either we can form a chain from *w*_1_ to *w* and then to *l*, or a chain from *w*_*k*_ to *w* and then to *l*. This chain has length at least ⌊*diam*(*T*)/2⌋+⌈*diam*(*T*)/2⌉+1>*diam*(*T*), a contradiction. This shows that *ecc*_*T*_(*w*)=⌈*diam*(*T*)/2⌉ and concludes the proof.

### Proof of Theorem ?? (Upper bound Meth.2):

Consider a given instance (*T*,*T*^′^). Take any leaf of *T* and assign it as the root, and do the same for *T*^′^. Although we have assumed roots of degree at least two so far, we use this rooting only for our analysis in order fix a parent-child relationship between nodes. Let *Q* be an optimal sequence of operations turning *T* into *T*^′^. We may assume that *Q* first contracts every non-mixed edge, and our algorithm does the same. Therefore, we suppose that *T* and *T*^′^ contain no non-mixed edges. Assume for our purposes that whenever a contraction takes place in *Q* between a node *u* and a child *v*, the *u* node stays in the tree and *v* gets removed (here the notion of a child is in the rooted sense with respect to our rooting above). Also assume that when there is an extension splitting a node *u*, then the newly created node becomes a child of *u* and *u* retains the same parent. It is easily checked that this only alters the name of nodes and not the sequence itself.

Call an internal node *v* of *T* a *good child* if the edge between *v* and its parent is good. Note that *v* has a unique corresponding node in *T*^′^ which we denote *v*^′^ (i.e. *v*^′^ is the root of the same clade as the subtree rooted at *v*). Further, call *v* a *bad-good* child if *v* is a good child, but either the label of *v* differs from that of *v*^′^, or *v* is incident to at least one bad edge (yes, children are capable of being both bad and good). Note that every bad subtree of *T* is rooted at a bad-good child, and observe that here we say that a bad-good child *v* that is incident to only good edges is a particular case of a bad subtree (i.e. *v* just has the wrong label).

We already know that *δ*(*T*,*T*^′^) is at least the number of bad edges in *T* and *T*^′^. Let *Q*^′^ be the set of operations of *Q* that are either flips, or contraction of good edges. We argue that |*Q*^′^| is at least the number of bad-good children in *T*. To see this, let *v* be a bad-good child. Assume first that *v* is not incident to any bad edge. If we never flip *v* nor remove it by contracting its parent edge, then *Q* cannot transform *T* into *T*^′^, as *v* and its underlying clade remain present in every tree from *T* to *T*^′^, but with the wrong label (because a contraction not removing *v* cannot remove the *v* clade, and extensions can create clades but not remove them). So we may assume that *v* gets flipped or that its parent edge gets contracted. A flip must be in *Q*^′^ and, observing that at any point the parent edge of *v* must be good, a contraction removing *v* must also be in *Q*^′^. Assume instead that *v* is incident to at least one bad edge {*v*,*w*}, with *w* a child of *v*. If *v* is never flipped nor removed owing to a contraction of its parent edge, then at some point *w* must be flipped so that the {*v*,*w*} edge gets contracted. Otherwise, if *v* gets removed, then its parent edge was contracted, again implying the contraction of a good edge. Either cases imply an operation in *Q*^′^. Importantly, observe that the operations in *Q*^′^ identified above are all distinct, since each one implies a flip or a node removal of a node in a different bad subtree of *T*.

Now, let *T*_1_,…,*T*_*k*_ be the bad subtrees of *T* and *T*^′^, and for each *i*∈{1,…,*k*}, let *t*_*i*_ be the number of bad edges in *T*_*i*_. Further denote $b = \sum _{i=1}^k t_{i}$. Since bad subtrees form pairs, our arguments above imply that *Q*^′^ has at least *k*/2 operations (because |*Q*^′^| is at least the number of bad trees in *T*, which is half the number of bad subtrees). The contraction of bad edges plus the operations of *Q*^′^ show that *Q* has at least $\sum _{i = 1}^k t_i + k/2 = b + k/2$ operations. Our algorithm contracts *b* edges in total. To count the number of flips, take any bad subtree *T*_*i*_. Then *t*_*i*_≥*diam*(*T*_*i*_)−2 and the number of flips we perform is at most ⌈*diam*(*T*_*i*_)/2⌉−1=⌈(*diam*(*T*_*i*_)−2)/2⌉≤*t*_*i*_/2+1. Note that this also holds when *T*_*i*_ contains no bad edge. Therefore, the number of operations that we perform is at most $b + \sum _{i=1}^k (t_i/2 + 1) = 3b/2 + k$. Our approximation ratio is therefore $\frac {3b/2 + k}{b + k/2} \leq \frac {2b + k}{b + k/2} = 2$.

## Data Availability

The software implemented during the current study is available in the pylabeledrf repository, https://github.com/DessimozLab/pylabeledrf.

## Abbreviations

RF: Robinson-Foulds

## Footnotes

1 The radius of *T* is a well-known graph parameter and is defined as the minimum eccentricity of a node of *T*. In a tree, the radius turns out to be ⌈*d**i**a**m*(*T*)/2⌉.
